# Enhancing Physical Health in Patients with Severe Mental Disorder: Addressing Physical Multimorbidity

**DOI:** 10.1192/j.eurpsy.2024.1021

**Published:** 2024-08-27

**Authors:** I. Š. Filipčić, I. Filipčić

**Affiliations:** ^1^Department of psychiatry and psychological medicine, University Hospital Zagreb; ^2^Psychiatric Clinica Sveti ivan, Zagreb, Croatia

## Abstract

**Introduction:**

Over the last two decades, a growing volume of research has discovered a correlation between severe mental disorders (SMD) and early mortality. This is attributed to the elevated incidence of chronic physical illnesses s and multimorbidity, resulting in a reduction of life expectancy by 10-20 years. Individuals with SMD exhibit lower rates of prevention, diagnosis, and treatment for medical comorbidities when contrasted with the general population (GP).

**Objectives:**

The objective is to assess the prevalence of CPM and its impact on psychiatric treatment outcomes in individuals with SMD, and to propose preventive interventions to enhance physical health.

**Methods:**

This nested cross-sectional study enrolled 343 SSD patients and 620 GEP.

**Results:**

Individuals diagnosed with SMD encounter CPM earlier in life compared to the GP. Notably, individuals under 35 years old within the schizophrenia spectrum disorder have almost three times higher odds for experiencing CPM compared to their GP counterparts, a difference that is both clinically and theoretically significant. This disparity is especially pronounced among younger women, with the gap widening the younger the patient is in comparison to peers in the general population. CPM has been identified as a factor affecting the outcomes of psychiatric treatment.

**Conclusions:**

The treatment approach for SMD should be tailored to accommodate the diverse physical multimorbidity patterns of patients. It’s imperative for future research to delve into how CPM impacts the outcomes of SMD treatments. There’s a pressing need for detailed treatment guidelines addressing CPM in patients with SMD.

**Disclosure of Interest:**

None Declared

